# Indeterminate Cell Histiocytosis in Association with Acute Myeloid Leukemia

**DOI:** 10.1155/2010/569345

**Published:** 2010-06-21

**Authors:** Filipa Ventura, Teresa Pereira, Maria da Luz Duarte, Herlander Marques, Fernando Pardal, Celeste Brito

**Affiliations:** ^1^Dermatology and Venereology Department, Braga Hospital, Apartado 2242, 4701-965 Braga, Portugal; ^2^Oncology Department, Braga Hospital, 4701-965 Braga, Portugal; ^3^Pathology Department, Braga Hospital, 4701-965 Braga, Portugal

## Abstract

Indeterminate cell histiocytosis (ICH) is a rare proliferative disorder, in which the predominant cells share morphologic and immunophenotypic features from both Langerhans and non-Langerhans cell histiocytosis. We describe a 62-year-old man presenting a 2-month history of firm nodular lesions on the upper lip. Histopathology, immunohistochemical, and ultrastructural analysis showed typical findings of ICH. The patient was treated with thalidomide and almost complete regression of the lesions was reached within 7 months. Nevertheless, one month after remission, he developed an acute myeloid leukemia of the subtype monocytic leukemia (M5). The patient's condition rapidly worsened and he died due to a respiratory failure four weeks later. We present this case because apart of being rare it joins the effectiveness of thalidomide and the association with an acute monocytic leukemia. A review of the literature is made.

## 1. Introduction

Indeterminate cell histiocytosis (ICH) is a rare proliferative disorder, in which the predominant cells share morphologic and immunophenotypic features from both Langerhans and non-Langerhans cell histiocytosis. Clinically, most patients present multiple asymptomatic papulonodules with a colour variation from pink to brownish, covered by intact skin. Solitary lesions may also occur like soft nodules mainly on the trunk and arms. ICH lesions are usually restricted to the skin [1]. The major part of these cases have an indolent and benign course, not requiring aggressive treatment.

## 2. Case Report

**Figure 1:**
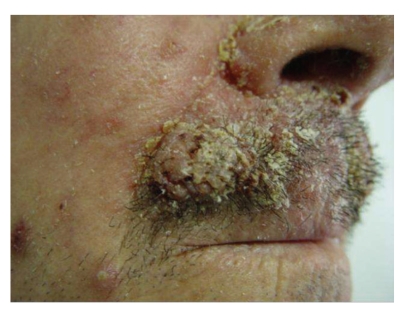
Nodular lesions located on the upper lip.

**Figure 2:**
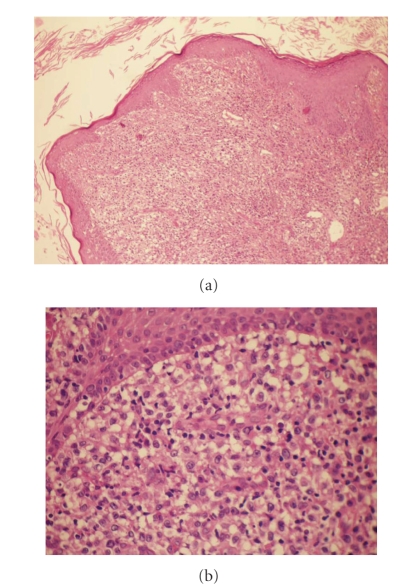
(a) Histopathology revealed a diffuse, monomorphous, nonepidermotropic infiltrate of large epithelioid cells throughout the entire dermis. (stain type—Hematoxylin and eosin; original magnification: ×100). (b) Histopathology revealed a diffuse, monomorphous, nonepidermotropic infiltrate of large epithelioid cells throughout the entire dermis. (stain type—Hematoxylin and eosin; original magnification: ×400).

**Figure 3:**
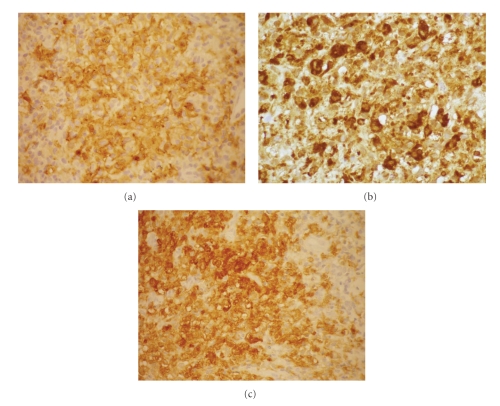
Immunohistochemical examination showed positivity for CD1a (a), CD68 (b), and S100 protein (c).

**Figure 4:**
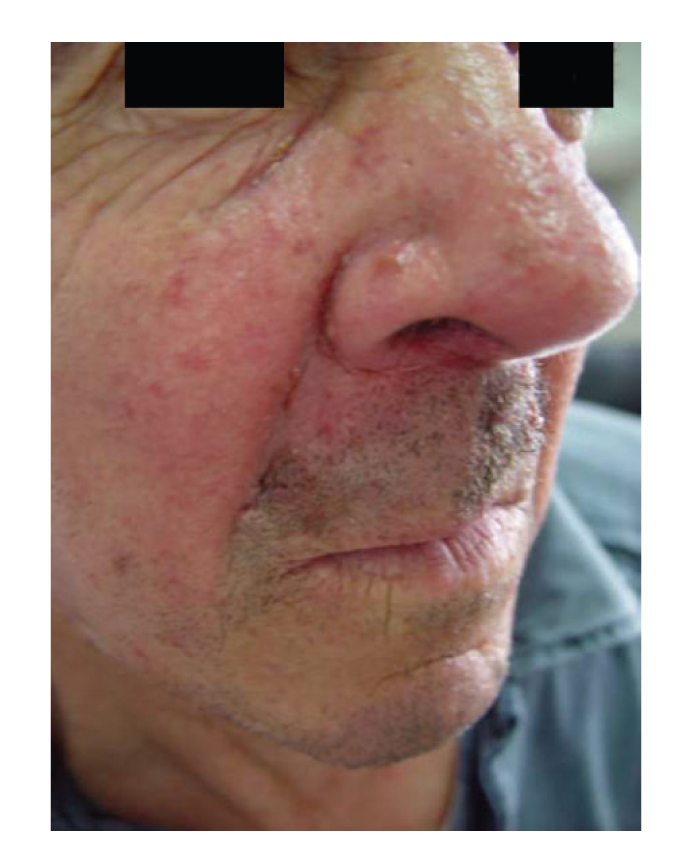
Regression of the lesions after 7 months of treatment with thalidomide.

A 62-year-old Caucasian man presented a 2-month history of firm nodular lesions on the upper lip (Figure 1). These lesions were isolated and confluent; some of them were covered by hemorrhagic crusts. The mucous membranes were not involved and the lesions were painless and nonpruritic. He was not taking any medication. Skin biopsy was taken and sent for histopathology and cultures. Histopathology showed a diffuse, monomorphous, nonepidermotropic infiltrate of large “histiocytic” cells with eosinophilic cytoplasm and irregularly shaped nuclei throughout the entire dermis. These cells were mixed with lymphocytes, plasma cells, and eosinophils (Figures 2(a) and 2(b)). Immunohistochemical examination revealed that the predominant cells coexpressed CD1a (Figure 3(a)), CD68 markers (Figure 3(b)), and S100 protein (Figure 3(c)). They were negative for CD3, CD4, CD8, CD20, CD30, CD79a, and FXIIIa markers. The ultrastructural analysis confirmed the dendritic morphology of the infiltrating cells and the absence of Birbeck granules. Culture for bacteria, mycobacteria, and fungi was negative. The diagnosis of ICH was made. Clinical staging, including complete hematologic examination with bone marrow biopsy, chest X-ray, and chest, abdominal and pelvic CT were normal. Thalidomide 200 mg daily was initiated after neurological examination. After 2 months of treatment, the lesions underwent gradual regression and the dose of thalidomide was reduced to 100 mg/day. Almost complete remission of the lesions was observed by the seventh month (Figure 4). No adverse effects from treatment were observed on the clinical and electrophysiological examinations. However, at this time, the laboratory findings showed hyperleucocytosis (white blood count 72,9 × 10^9^/L) with 67% blasts, anemia (hemoglobin 6,1 g/dL), thrombocytopenia (platelet count 34 × 10^9^/L), hyperuricemia (serum acid uric 12,4 mg/dL, normal), high levels of lactate dehydrogenase (1087 U/L, normal), and *β*_2_-microglobulin (2610 ng/mL, normal). The bone marrow aspirate was hypercellular with extensive infiltrate by the blasts. The blasts were positive for *α*-naphthylacetate esterase, myeloperoxidase, and Periodic Acid-Schiff (PAS). Flow cytometry analysis revealed a blast population with monocytic differentiation expressing CD-11B, CD14, CD15, CD33, CD36, CD45, CD56, CD64, and HLA-DR, but lacking CD34 expression. These findings support a diagnosis of an acute monocytic leukemia by the World Health Organization (WHO). Cytogenetic analysis showed two abnormal clones on the chromosomes 7 and 18. Treatment with hydroxyurea had been initiated; however patient's condition deteriorated and he died 1 month later due to a respiratory failure.

## 3. Discussion

Histiocytoses are a heterogeneous group of disorders characterized by the proliferation and accumulation of cells of the mononuclear-macrophage system and the dendritic cells [2]. All these cells derive from neutrophil/macrophage lineage progenitor cells in bone marrow [3, 4]. There are reported in the literature different opinions trying to classify these challenging but poorly understood disorders. 

In what concerns to the indeterminate cells' origin and the ICH there is still a lot of controversy. Indeterminate cells are defined to be closely related to Langerhans cells, histologically and immunocytochemically, but do not contain Birbeck granules. Some authors have reported that indeterminate cells may represent precursors of Langerhans cells which acquire Birbeck granules as they transit from dermal to epidermal sites, possibly the result of the interaction between their cell receptors and epidermis-specific ligands [1, 2, 5]. Others suggest that indeterminate cells represent members of the dermal/epidermal dendritic cell system that migrate from the skin to the regional lymph nodes [1, 2, 5]. Hence ICH seems to be a disorder of proliferating indeterminate cells which have been locally arrested before they can leave the skin travelling as veiled cells via the lymphatics to the T-cell-dependent paracortical areas of the regional lymph nodes [1, 2, 5]. 

More recently, it has been suggested that what today is described as ICH could be a variant of non-Langerhans' cell histiocytosis (NLCH) rather than an overlap between Langerhans cell histiocytosis (LCH) and NLCH [2]. In fact ICH shows mainly features of NLCH except for the consistent expression of S-100 protein. This is not totally surprising because aberrant S-100 protein has been reported before [2]. Other authors believe that there are disorders of Langerhans cells and macrophages; both differ according to the time cycle of the lesions. Late stages of LCH may completely lose Langerhans cells markers and then consist mainly of xanthomatized macrophages. On the other hand, macrophage disorders have a polymorphous presentation which also includes their immunoprofile.

Nowadays it is not clear that ICH is a separate entity or if represents various macrophage disorders identified at different periods in the inflammatory response.

Proliferative disorders of indeterminate cells have a wide spectrum of clinical severity and a variable clinical course. The majority of cases have a benign clinical behaviour and self-healing skin lesions that completely or partially regress spontaneously, requiring no aggressive treatments [5, 6]. Chemotherapy is necessary for aggressive cutaneous ICH, defined as ICH involving over 50% of the body surface and lasting at least 6 months with no tendency to spontaneous resolution and with new crops of lesions continuing to appear [6]. In our case in spite of localized lesions without visceral involvement, it was a disfigurating cutaneous lesion and we have decided to treat the patient with thalidomide 200 mg/day, with almost complete regression of the lesions after 7 months. Our decision was based on the predominantly cutaneous pharmacological effect, immunomodulatory and anti-inflammatory properties of this drug. It has also been shown that thalidomide modulated the function properties of monocyte-derived dendritic cells [7]. 

An association between ICH and malignancies has been described in four patients: twice with low grade B-cell lymphomas [2], once with mast cell leukemia [8], and another with myelomonocitic leukemia [1]. Our patient presented a monocytic leukemia 7 months after the onset of the skin lesions.

While antineoplastic drugs are used, such as cyclophosphamide, vinblastine, etoposide, and 2-chlorodeoxyadenosine, to treat aggressive diffuse forms of ICH, the possibility of this treatment being related on the etiology of oncohematological disorders cannot be excluded [6]. It does not occur in our case because it was used thalidomide, with proven effectiveness on multiple myeloma and myelodysplastic syndrome [9].

The biological basis of the association between an ICH and leukemia remains unknown. It is known that macrophages are derived from circulating monocytes and thus share a common bone-marrow progenitor cell, the neutrophil/macrophage colony-forming unit. So, in what concerns to our case, the association between indeterminate cell histiocytosis and monocytic leukemia was probably greater than that by chance alone. We present this case because apart of being rare it joins the effectiveness of thalidomide and the association with an acute monocytic leukemia.
